# Effect of beach chair position on hemodynamics and cardiovascular events in patients undergoing shoulder arthroscopy for rotator cuff injury combined with shoulder adhesion

**DOI:** 10.3389/fsurg.2026.1687643

**Published:** 2026-08-11

**Authors:** Yuxin Zhang, Ling Zhao, Jidong Lv, Jie Lei

**Affiliations:** 1 Anaesthesiology, Jiashan First People's Hospital Maternal and Child Health Hospital, Zhejiang, Jiashan, China; 2 Anaesthesiology, Jiashan County Maternal and Child Health Hospital, Zhejiang, Jiashan, China

**Keywords:** beach chair position, cardiovascular events, hemodynamics, rotator cuff injury, shoulder adhesion, shoulder arthroscopy

## Abstract

**Objective:**

To investigate the effect of beach chair position on hemodynamics and cardiovascular events in patients with rotator cuff injury combined with shoulder adhesion who underwent shoulder arthroscopy, and to provide a theoretical basis for rational placement of surgical position in clinical practice.

**Methods:**

We conducted a retrospective analysis of 148 patients (BC group: *n* = 78; LD group: *n* = 70) who underwent shoulder arthroscopy between February 2021 and December 2024. Data were extracted from electronic medical records. Baseline characteristics including operator experience and duration of position maintenance were collected. Surgical parameters (operation time, intraoperative blood loss, urine output, fluid administration), hemodynamic changes measured at baseline, 5 min after positioning, and every 15 min thereafter (differences in systolic/diastolic blood pressure and heart rate before/after positioning), shoulder function recovery (UCLA and ASES scores), range of motion (forward flexion, external rotation, abduction), and cardiovascular complications (arrhythmias requiring intervention defined as episodes lasting >5 min with hemodynamic compromise, myocardial infarction, angina, hypotension) were compared between groups.

**Results:**

Comparison of operative time, intraoperative bleeding, intraoperative urine output, intraoperative fluid administration, pre- and post-operative UCLA scores, ASES scores, anterior flexion mobility, external rotation mobility, and abduction mobility between patients in the two groups showed no statistically significant differences (*P* > 0.05). The difference in systolic blood pressure, diastolic blood pressure and heart rate before and after surgical position placement of patients in the beach chair position group was higher than that of patients in the lateral decubitus position group, and the incidence of cardiovascular events such as cardiac arrhythmia, myocardial infarction, angina pectoris, hypotension and postural hypotension was higher than that of patients in the lateral decubitus position group, and the difference was statistically significant (*P* < 0.05).

**Conclusion:**

The beach chair position for shoulder arthroscopy in patients with rotator cuff injury combined with shoulder adhesion is comparable to the lateral decubitus position in terms of surgical efficiency and surgical trauma, and similar to the lateral decubitus position in terms of improving shoulder function and mobility, but it may cause hemodynamic fluctuations and increase the risk of cardiovascular events. Clinical decision-making should prioritize position selection based on specific cardiovascular risk factors, with the lateral decubitus position recommended for patients with pre-existing cardiovascular disease, uncontrolled hypertension, or age over 70 years.

## 1 Introduction

Rotator cuff injury is one of the most common diseases causing periapical pain and shoulder dysfunction, and it is commonly seen in older people over 60 years of age, and the prevalence increases accordingly with gradual increase in age, accounting for about 30% of older people over 60 years of age and 60% of older people over 80 years of age (1). Symptoms of rotator cuff injury include pain, weak force, limited activity and intra-articular abnormal sound, which often occurs in repetitive sports requiring extreme abduction of the shoulder joint (such as freestyle, backstroke, butterfly, baseball, weight lifting, racket sports, etc.), and those who are not treated in a timely manner may suffer from shoulder instability or secondary arthrocontracture, which leads to joint dysfunction and seriously affects the patients' quality of life (2). Rotator cuff injury patients are often affected by inflammatory response, joint instability and other factors, shoulder joint adhesion, rotator cuff injury itself will lead to inflammatory response, and shoulder joint adhesion further exacerbates this inflammation, the formation of fibrous scar tissue, so that the joint capsule and the surrounding tissues are adhesive, limiting joint activities, these two conditions affect each other, a vicious cycle, which makes it even more difficult to restore the function of the shoulder joint (3). Therefore, it is of great significance to take timely and effective therapeutic measures for rotator cuff injury combined with adhesions in the shoulder joint.

Shoulder arthroscopic surgery is a new type of clinical surgery, mainly for the treatment of periarticular tissues and injuries of the shoulder joint, which has the advantages of small incision, low trauma, and fast postoperative recovery, and has been commonly used in clinical practice (4). Jaramillo Quiceno GA et al. (5) proposed that patients with preoperative mild shoulder adhesion were treated with rotator cuff suture combined with arthroscopic release and manipulation. A study by de Sa R et al. (6) demonstrated that patients with rotator cuff injuries combined with shoulder adhesions had significant improvement in shoulder function and pain after arthroscopic acromial decompression. Thus, shoulder arthroscopy may have good efficacy in the treatment of rotator cuff injury combined with shoulder adhesion. In the process of treatment, the overall efficacy and prognosis are affected to a certain extent by the patient's position, intra-articular pressure wash fluid and hemodynamics and other factors, and with the age of the body, its own physical and joint tissues will decline to varying degrees, and the face of the surgical operation is very susceptible to injury (7). Some studies have pointed out that choosing the appropriate position for patients during surgical treatment can provide protection for their prognosis and therapeutic efficacy, and reduce the impact on hemodynamics by adopting an effective position (8). Therefore, it is necessary to analyze the adoption of reasonable and effective positions during surgery, and select effective positions to improve the efficacy and guarantee the prognosis. The beach chair position is widely used in shoulder arthroscopy, which has the advantage of facilitating surgical operation, less bleeding, and is conducive to traction and checking the anatomical position of the shoulder joint (9). However, Meta F et al. (10) found that the beach chair position has a greater impact on the hemodynamics of patients, especially elderly hypertensive patients, whose own circulation and cerebral vascular regulation is poorer, and general anesthesia in the head-height-sole position has obvious circulatory disturbances in elderly patients, with the risk of cerebral ischemia and hypoxia. At present, the clinical research on the use of beach chair position for rotator cuff injury combined with shoulder joint adhesion in patients undergoing shoulder arthroscopy is relatively limited.

Based on this, this paper analyzes the effect of beach chair position on hemodynamics and cardiovascular events in patients undergoing shoulder arthroscopy for rotator cuff injury combined with shoulder adhesion by establishing a retrospective cohort study, which is expected to help doctors choose a more reasonable surgical position, provide a reference to formulate a safer and more effective shoulder arthroscopy plan, improve the safety of shoulder arthroscopy in patients with rotator cuff injury combined with shoulder adhesion, and reduce the cardiovascular risk, and improve patient prognosis.

## 2 Materials and methods

### 2.1 Statement of ethics

This study was approved by our Institutional Review Board and Ethics Committee (Approval Number: JSFY-2021-035). Given that this study was retrospective and only de-identified patient data were used, informed consent was not required as there was no risk or adverse effect on patient care. This waiver is in line with regulatory and ethical guidelines related to retrospective studies.

### 2.2 Study design

This retrospective analysis studied 148 patients with rotator cuff injuries combined with shoulder adhesions who underwent shoulder arthroscopy at our institution between February 2021 and December 2024 as the study population, with patient data obtained from the medical record system, and were divided into a lateral decubitus position group (*n* = 70) vs. a beach chair position group (*n* = 78) based on the different surgical positions. As inherent to retrospective studies, there is a potential risk of selection bias. To minimize this, we applied strict inclusion and exclusion criteria and verified that baseline characteristics were comparable between groups. The surgical position was determined by the attending surgeon based on institutional practice and surgeon preference, which was documented in the operative notes.

### 2.3 Sample size determination

A *post-hoc* power analysis was performed using the primary outcome of cardiovascular event rate. With a cardiovascular event rate of 1.43% in the lateral decubitus position group and 8.97% in the beach chair position group, alpha set at 0.05, the study achieved a statistical power of 0.82, indicating adequate power to detect clinically significant differences in cardiovascular events between the two groups. The sample size was determined based on the available patient records during the study period from our institution.

### 2.4 Inclusion criteria

Inclusion criteria: (1) Meet the criteria of rotator cuff injury combined with adhesion in rotator cuff tear combined Chinese and Western medicine diagnosis and treatment guidelines (11): ① Shoulder joint pain, weakness, preoperative shoulder MRI or ultrasound and other imaging tests show and confirm the existence of rotator cuff injury under intraoperative arthroscopy; ② Shoulder active and passive mobility to meet the following criteria: anterior flexion ≤ 90°, abduction ≤ 90°, external rotation ≤ 35°, internal rotation ≤ great trochanteric level (when measuring internal rotation, the patient was asked to hold the palm of the hand backward and abduct the thumb, and the position where the tip of the thumb could be touched was taken as the sign of internal rotation), and the diagnosis could be confirmed with the two items mentioned above. (2) Age ≥18 years old; (3) 3 months of conservative treatment was ineffective; (4) Shoulder arthroscopy was accepted; and (5) Clinical data were complete.

Exclusion criteria: (1) cases with huge rotator cuff tear (>5 cm), combined with injury to the infraspinatus or subscapularis tendon, injury to the superior glenoid labrum from anterior to posterior tear, injury to the anterior inferior shoulder capsule-glenoid labrum complex, and instability of the shoulder joint; (2) cases with severe glenohumeral arthritis; (3) cases with acromioclavicular arthritis requiring distal clavicle resection; (4) cases requiring tendonectomy of the biceps long head tendon or immobilization; (5) cases of revision; and (6) cases with severe osteoporosis.

### 2.5 Anesthetic management

All surgeries were performed under general anesthesia assisted by cervical plexus nerve block. General anesthesia was induced with propofol (1.5–2.5 mg/kg), fentanyl (2–3 μg/kg), and rocuronium (0.6–1.0 mg/kg) for endotracheal intubation. Anesthesia was maintained with sevoflurane (1.5%–2.5%) and remifentanil (0.1–0.3 μg/kg/min). Intraoperative monitoring included continuous electrocardiography, noninvasive blood pressure measurement every 3 min, pulse oximetry, end-tidal carbon dioxide monitoring, and urine output. Mean arterial pressure was maintained at 65–90 mmHg throughout the procedure. If hypotension (systolic blood pressure <90 mmHg or mean arterial pressure <65 mmHg) occurred, it was treated with fluid boluses and/or vasopressors (phenylephrine 50–100 μg or ephedrine 5–10 mg). If bradycardia (heart rate <50 beats/min) occurred, atropine 0.5 mg was administered. All patients received standardized fluid management with crystalloid infusion at 5–8 mL/kg/h, with additional boluses administered based on hemodynamic parameters and urine output.

### 2.6 Surgical and positional approach

The lateral decubitus group was placed in the lateral decubitus position, and the beach chair group was fixed on the operating table in the beach chair position with the backrest elevated to 70–80 degrees. The mean duration of position maintenance was 98.4 ± 12.6 min in the beach chair position group and 97.8 ± 11.9 min in the lateral decubitus position group. All procedures were performed by surgeons with a minimum of 5 years of experience in shoulder arthroscopy (mean experience: 8.2 ± 2.4 years in the beach chair position group and 7.9 ± 2.1 years in the lateral decubitus position group; *P* = 0.468). Skin traction was applied to the affected limb using a traction frame. Rotator cuff suture surgery was performed with arthroscopic posterior approach for observation, anterior approach and 2 lateral approaches. The glenohumeral joint was explored first, and arthroscopic capsular release was performed by alternating between anterior and posterior approaches to loosen the anterior, posterior, inferior, and superior joint capsule with a basket clamp or radiofrequency, respectively. Subsequently, the biceps long head tendon, glenoid labrum, articular cartilage and subscapularis muscle were explored and the corresponding problems were treated. Subsequently, a subacromial working channel is established, the subacromial bursa is cleaned, and acromioplasty is performed in patients with subacromial impingement syndrome. The type of rotator cuff injury is assessed, appropriate release is performed and scarred tissue is removed. After grinding away the hyperplastic bone on the surface of the greater tuberosity, the inner row of the greater tuberosity was placed immediately adjacent to the cartilage margin, and one or two inner row anchors with wires were placed according to the size of the rupture, and the rotator cuff was secured to the surface of the greater tuberosity in a wire bridge fashion with external rows of nails after all the sutures were tied through the tendon breaks separately. Postoperatively, all patients wore a 30° abduction and mild external rotation brace for 6 weeks after surgery. Passive shoulder joint activities were performed 3 times a day under the principle of painlessness from the first week after surgery, and muscle strength exercises were gradually performed at 6 weeks after surgery.

### 2.7 General information collection

The general data of patients were retrospectively collected through the electronic medical record system, which mainly included gender, age, body mass index, smoking history, drinking history, co-morbidities [hypertension in accordance with the 《The Japanese Society of Hypertension Guidelines for Self-monitoring of Blood Pressure at Home (Second Edition)》 (12) diagnostic criteria for hypertension, diabetes mellitus meeting the 《Application of the Chinese Expert Consensus on Diabetes Classification in clinical practice》 (13) Diagnostic Criteria for Diabetes Mellitus), American Society of Anesthesiologists (ASA) classification (14), disease duration, site of disease, operator experience (defined as the number of years performing shoulder arthroscopy procedures), and duration of position maintenance (defined as the total time from initial positioning to position release at the end of surgery).

### 2.8 Collection of surgical indexes

The surgical indexes of the two groups of patients were retrospectively collected through the electronic medical record system, which mainly contained surgical time, intraoperative bleeding volume, intraoperative urine output and intraoperative fluid administration volume.

### 2.9 Hemodynamic indexes

Hemodynamic parameters were recorded at the following standardized time points: T0 (baseline, immediately before positioning), T1 (5 min after position placement), T2 (15 min after positioning), T3 (30 min after positioning), and subsequently every 15 min until the end of surgery. The systolic blood pressure, diastolic blood pressure and heart rate values recorded in the electrocardiographic monitoring equipment before and after the placement of the patients in the surgical position were retrospectively collected through the electronic medical record system, and the difference between the T0 and T1 values was calculated as the change in systolic blood pressure, diastolic blood pressure and heart rate, respectively, representing the acute hemodynamic response to positioning.

### 2.10 Cardiovascular event definitions and assessment

Cardiovascular events were assessed and documented by the anesthesiologist and attending surgeon during the intraoperative and perioperative period. The following objective diagnostic criteria were used: Arrhythmia requiring intervention was defined as any new-onset irregular heart rhythm documented on continuous electrocardiographic monitoring that met at least one of the following criteria: (a) atrial fibrillation or atrial flutter with rapid ventricular response (heart rate >150 beats/min) lasting more than 5 min; (b) ventricular tachycardia (≥3 consecutive ventricular beats at rate >100 beats/min) of any duration; (c) symptomatic bradycardia (<50 beats/min) lasting more than 5 min with associated hypotension (systolic blood pressure <90 mmHg) or signs of inadequate perfusion; (d) tachycardia (>120 beats/min) lasting more than 5 min with hemodynamic compromise defined as systolic blood pressure decrease >20% from baseline; or (e) any arrhythmia requiring pharmacological intervention (antiarrhythmic agents, atropine for bradycardia) or electrical cardioversion. Myocardial infarction was diagnosed based on elevated cardiac troponin levels (troponin I > 0.04 ng/mL, measured when clinically indicated) combined with clinical symptoms (chest pain, dyspnea) and/or electrocardiographic changes (ST-segment elevation ≥1 mm in two contiguous leads, ST-segment depression ≥0.5 mm, new Q waves ≥0.03 s in width, or new T-wave inversions ≥1 mm). Angina pectoris was defined as chest pain or discomfort consistent with myocardial ischemia lasting more than 5 min, with or without electrocardiographic changes, that resolved with rest or nitrate administration. Hypotension was defined as systolic blood pressure <90 mmHg or mean arterial pressure <65 mmHg persisting for more than 5 min and requiring pharmacological intervention with vasopressors. Postural hypotension was defined as a decrease in systolic blood pressure ≥20 mmHg or diastolic blood pressure ≥10 mmHg within 3 min of position change that was symptomatic (dizziness, lightheadedness, visual disturbances) or required intervention (fluid bolus or vasopressor administration).

### 2.11 Shoulder function

The shoulder scores of The University of California-Los Angeles (UCLA) and the rating scale of the American Shoulder and Elbow Surgeons (ASES) were retrospectively collected from the electronic medical record system for the two groups of patients in the preoperative and postoperative periods of 3 months. The UCLA scale evaluates the shoulder joint from the aspects of pain, function, active forward flexion mobility, anterior flexion strength, and patient satisfaction, with a total score of 35 points, and the higher the score indicates the better the shoulder joint function of the patient (15). The ASES scale evaluates the shoulder joint function from the 3 aspects of pain, stability, and function, with a total score of 100 points, of which pain accounted for 36% of the total score, stability accounted for 36% of the total score, and function accounted for 28% of the total score, with higher scores indicating better shoulder joint function in patients (16).

### 2.12 Shoulder mobility

Anterior flexion mobility, external rotation mobility and abduction mobility were retrospectively collected through the electronic medical record system in the two groups of patients. The patients took the standing position or sitting position, with the shoulder joint in the neutral position, flexed the shoulder joint forward, and lifted the arm to the maximum extent as much as possible, and the measurer used a protractor to measure the angle between the upper arm and the torso that is, the degree of anterior flexion mobility; the patients stood or sat in the standing or sitting position, with the shoulder joint in the neutral position, the elbow joint in 90-degree flexion, and the forearm kept in the neutral position, externally rotate the forearm, try to move the back of the hand to the lateral side, and the measurer uses a protractor to measure the angle between the forearm and the trunk as the degree of externally rotated activity; the patient stands or sits with the shoulder joint in the neutral position, abducts the shoulder joint, and tries to raise the arm to the side, and the measurer uses a protractor to measure the angle between the upper arm and the trunk as the degree of abducted activity.

### 2.13 Incidence of cardiovascular events

The incidence of cardiovascular events such as arrhythmia, myocardial infarction, angina pectoris, hypotension and postural hypotension were retrospectively collected in both groups through the electronic medical record system.

### 2.14 Statistical methods

SPSS25.0 statistical software was used to analyze the data, and the counting information was expressed as the number of cases (percentage), and the *χ*^2^ test was adopted; the measurement information conforming to normal distribution was expressed as (x̅ ± s), and the t test was adopted. The difference was considered statistically significant at *P* < 0.05.

## 3 Results

### 3.1 Comparison of general information of patients in two groups

Comparison of the general information of the two groups of patients, such as gender, age, body mass index, smoking history, drinking history, co-morbidities, ASA classification, duration of the disease, location of the disease, operator experience, and duration of position maintenance, the difference is not statistically significant (*P* > 0.05), suggesting that the groups are comparable, see Table 1.

**Table 1:** Comparison of general information of the two groups of patients.

Index	Category	Lateral decubitus position group (*n* = 70)	Beach chair position group (*n* = 78)	*χ*²/t value	*P* value
Sex (*n*)	Male	42	48	0.037	0.848
	Female	28	30		
Age (years)		65.34 ± 4.68	65.60 ± 4.72	0.336	0.737
Body mass index (kg/m²)		21.70 ± 3.42	22.04 ± 3.50	0.596	0.552
Smoking history (*n*)		15	18	0.058	0.810
Drinking history (*n*)		10	14	0.364	0.546
Complication (*n*)	Hypertension	8	12	0.494	0.482
	Diabetes	4	7	0.570	0.450
ASA level (*n*)	Level I	39	45	0.059	0.808
	Level II	31	33		
Course of disease (months)		8.70 ± 1.46	8.92 ± 1.50	0.902	0.368
Disease area	Left side	37	39	0.121	0.728
	Right side	33	39		
Operator experience (years)		7.9 ± 2.1	8.2 ± 2.4	0.824	0.411
Duration of position maintenance (min)		97.8 ± 11.9	98.4 ± 12.6	0.298	0.766

### 3.2 Comparison of surgical indexes between the two groups

**Figure 1:**
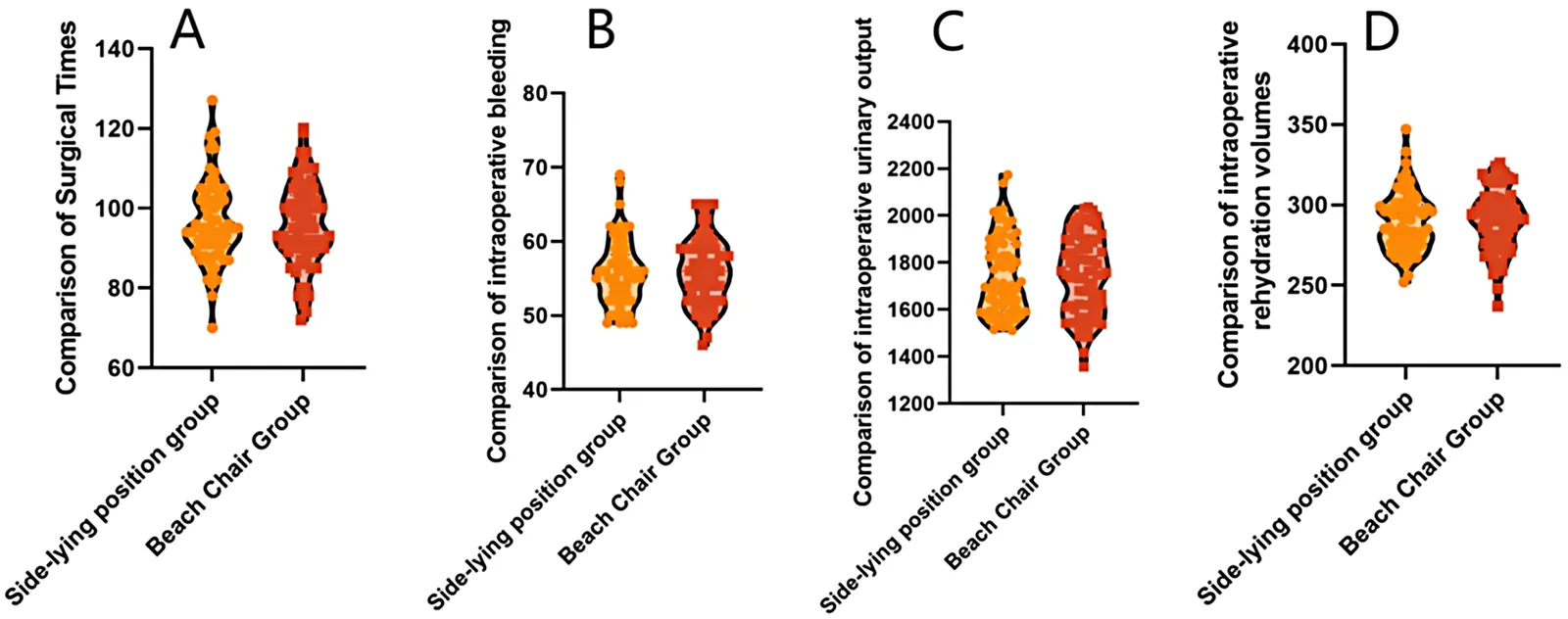
Comparison of surgical indexes between the two groups of patients. **(A)** Comparison of surgical times; **(B)** Comparison of intraoperative bleeding; **(C)** Comparison of intraoperative urinary output; **(D)** Comparison of intraoperative.

Surgical time is used to assess surgical efficiency, intraoperative bleeding volume is used to assess surgical risk, intraoperative urine output and intraoperative fluid administration volume are used to assess the fluid balance status, the shorter the surgical time, the lower the intraoperative bleeding volume, the reasonable intraoperative urine output and intraoperative fluid administration volume, which suggests that the higher the efficiency of the surgery, the lower the risk of the surgery, and the balanced fluid status. In the two groups, the operation time (96.58 ± 10.24 vs. 95.80 ± 10.35) min, intraoperative bleeding (55.74 ± 4.36 vs. 55.92 ± 4.40) mL, intraoperative urine output (1,742.86 ± 165.44 vs. 1,747.25 ± 166.28) mL, and intraoperative fluid administration volume (289.35 ± 18.35 vs. 290.14 ± 18.62) mL were compared, and the differences were not statistically significant (*P* > 0.05), see Figure 1.

### 3.3 Comparison of hemodynamic indexes between the two groups of patients

**Figure 2:**
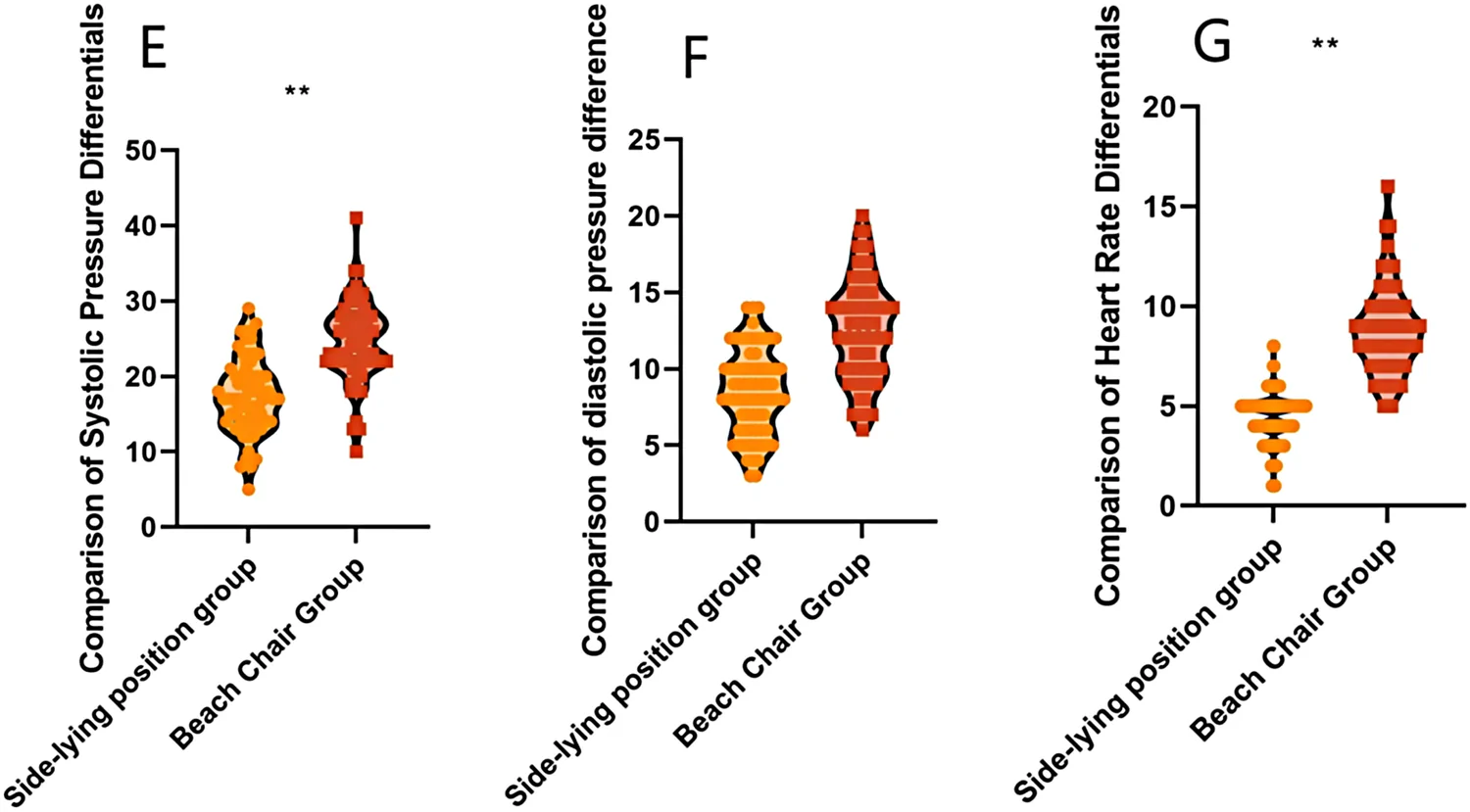
Comparison of hemodynamic indexes before and after position change in the two groups of patients. **(A)** Comparison of systolic pressure differentials; **(B)** Comparison of diastolic pressure difference; **(C)** Comparison of heart rate differentials.

Systolic blood pressure difference, diastolic blood pressure difference and heart rate difference are used to assess the hemodynamic changes of the body, and the higher the indexes, the greater the hemodynamic fluctuations before and after the position change. The systolic blood pressure difference (24.60 ± 5.12 vs. 17.34 ± 4.90) mmHg, diastolic blood pressure difference (12.54 ± 3.25 vs. 8.50 ± 2.78) mmHg, and heart rate difference (8.76 ± 2.14 vs. 4.38 ± 1.25) beats/min in the beach chair group were higher than that of the lateral decubitus position group, and the differences were statistically significant (*P* < 0.05), suggesting that compared with the lateral decubitus position, the beach chair position has a greater hemodynamic impact on patients undergoing shoulder arthroscopy for rotator cuff injuries combined with shoulder adhesions, see Figure 2.

### 3.4 Comparison of preoperative and postoperative UCLA and ASES scores between two groups of patients

UCLA and ASES scores are used to assess shoulder joint function, and the higher the two scores, the better the shoulder joint function. Preoperatively, the UCLA score and ASES score of the two groups were compared, and the difference was not statistically significant (*P* > 0.05), see Table 2.

**Table 2:** Comparison of preoperative UCLA and ASES scores between the two groups (x̅ ± s, points).

Index	Time	Lateral decubitus position group (*n* = 70)	Beach chair position group (*n* = 78)	*t* value	*P* value
UCLA scores	Preoperative	7.85 ± 1.76	7.74 ± 1.65	0.392	0.695
ASES scores	Preoperative	31.12 ± 2.58	30.96 ± 2.62	0.374	0.709

**Figure 3:**
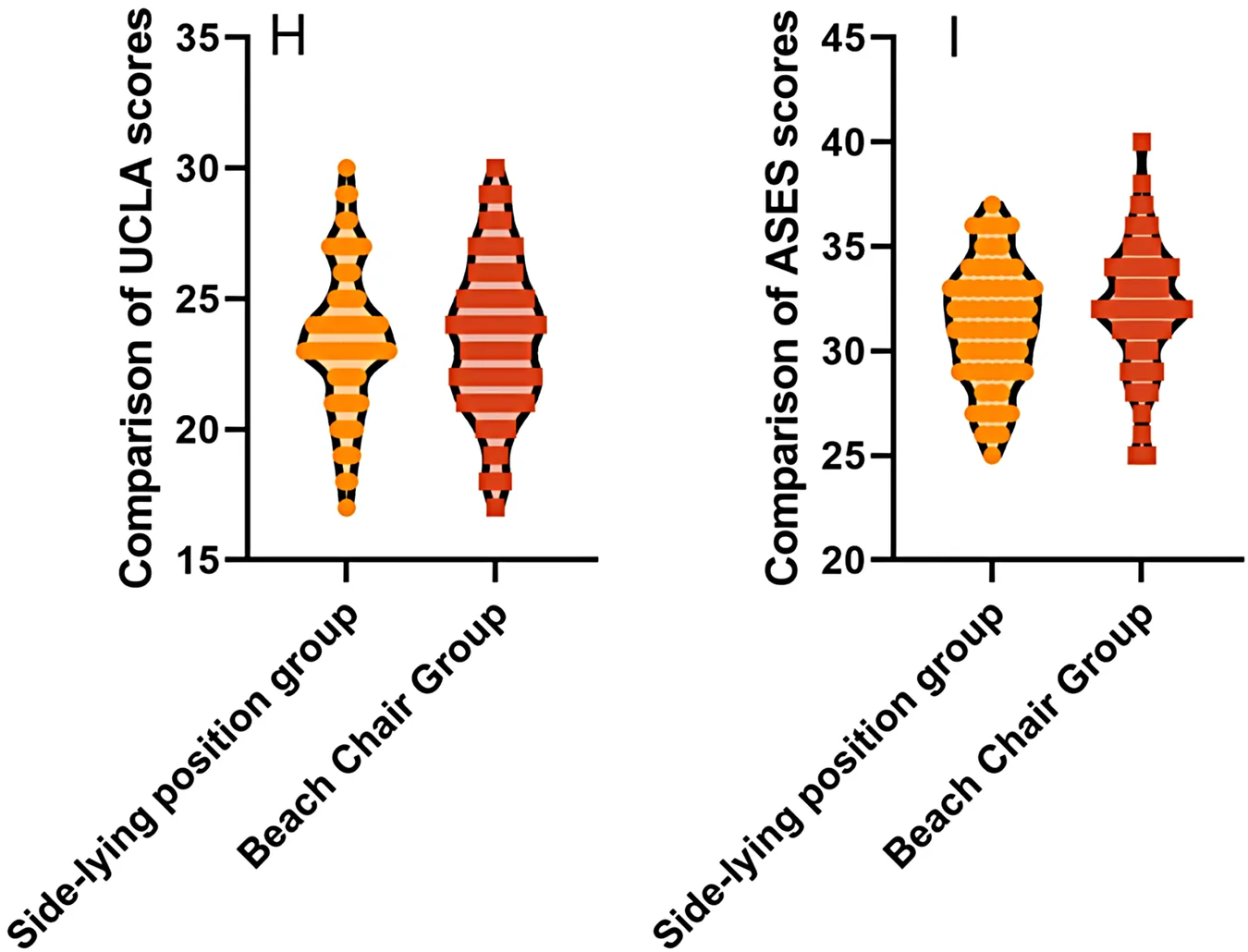
Comparison of postoperative UCLA and ASES scores between the two groups of patients. **(A)** Comparison of UcLA scores; **(B)** Comparison of ASES scores.

At 3 months after surgery, the UCLA score (23.46 ± 2.77 vs. 23.54 ± 2.90) and ASES score (31.25 ± 2.86 vs. 32.05 ± 2.94) of patients in the two groups were compared, and the differences were not statistically significant (*P* > 0.05), suggesting that the beach chair position on the shoulder function of patients with rotator cuff injuries combined with adhesions of shoulder joints who underwent arthroscopy of the shoulder was comparable to the lateral decubitus position. The improvement effect is comparable to that of the lateral decubitus position, see Figure 3.

### 3.5 Comparison of preoperative and postoperative shoulder joint mobility between the two groups of patients

Anterior flexion mobility, external rotation mobility, and abduction mobility were used to assess shoulder joint mobility, and the higher the indexes, the more ideal the shoulder joint mobility. Preoperatively, the difference between the two groups was not statistically significant (*P* > 0.05) when comparing the anterior flexion mobility (54.33 ± 5.78 vs. 54.10 ± 5.85)°, external rotation mobility (12.56 ± 2.14 vs. 12.44 ± 2.20)°, and abduction mobility (52.15 ± 5.40 vs. 52.46 ± 5.58)°, as shown in Table 3.

**Table 3:** Comparison of preoperative shoulder mobility between the two groups (x̅ ± s).

Index	Time	Lateral decubitus position group (*n* = 70)	Beach chair position group (*n* = 78)	*t* value	*P* value
Forward flexion mobility (°)	Preoperative	54.33 ± 5.78	54.10 ± 5.85	0.240	0.811
External rotation activity (°)	Preoperative	12.56 ± 2.14	12.44 ± 2.20	0.336	0.738
Abduction activity level (°)	Preoperative	52.15 ± 5.40	52.46 ± 5.58	0.343	0.732

**Figure 4:**
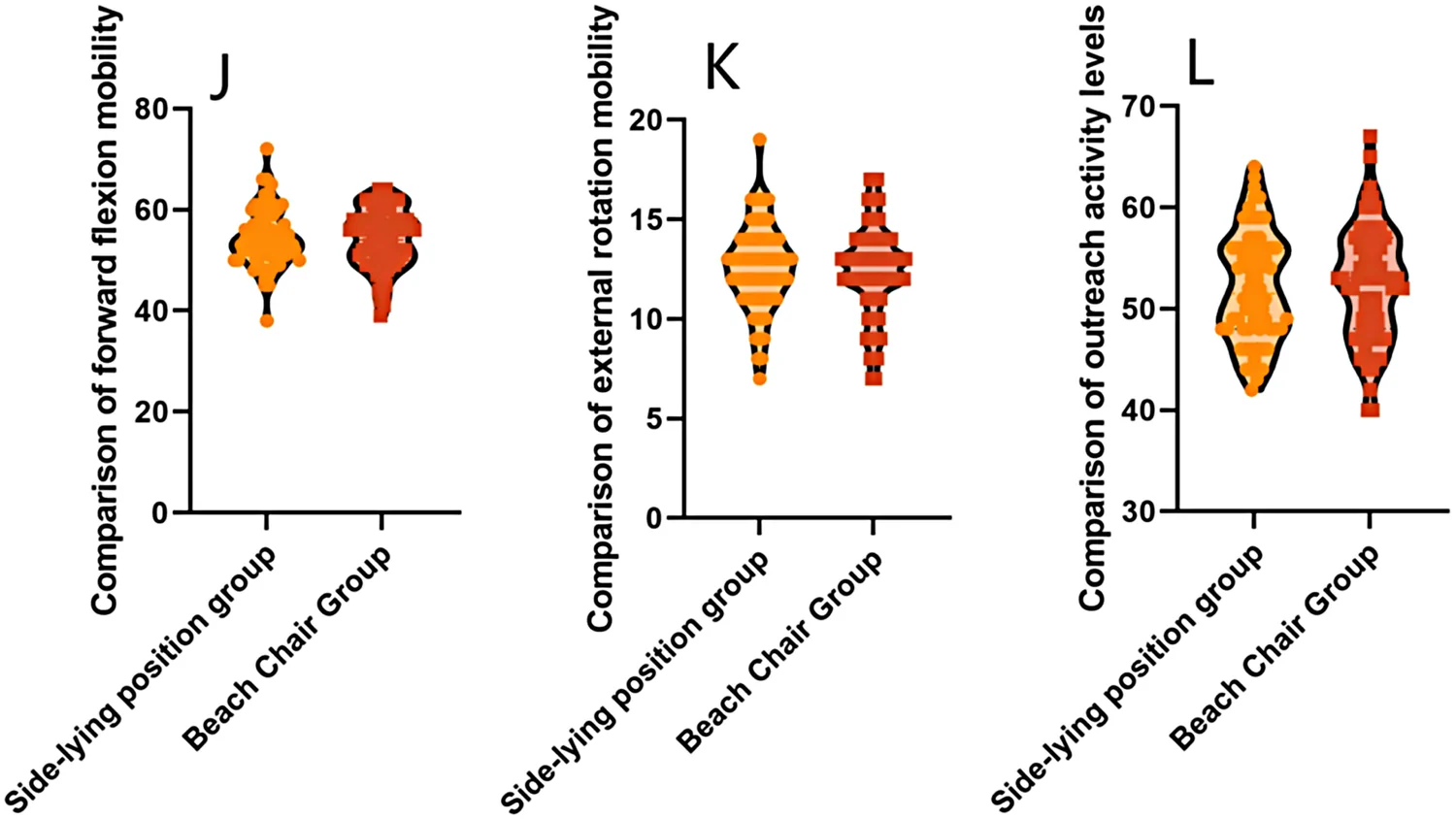
Comparison of postoperative shoulder mobility between the two groups of patients. **(A)** Comparison of forward flexion mobility; **(B)** Comparison of external rotation mobility; **(C)** Comparison of outreach activity levels.

At 3 months after surgery, when comparing the anterior flexion mobility (134.58 ± 14.26 vs. 135.05 ± 14.40)°, external rotation mobility (33.27 ± 3.80 vs. 33.45 ± 3.92)°, and abduction mobility (123.58 ± 12.76 vs. 123.20 ± 12.95)° between the two groups of patients, the differences were not statistically significant (*P* > 0.05), suggesting that the beach chair position is comparable to the lateral decubitus position in improving shoulder joint mobility in patients with rotator cuff injury combined with shoulder adhesion undergoing shoulder arthroscopy, see Figure 4.

### 3.6 Comparison of cardiovascular event rate between two groups of patients

The cardiovascular event rate is used to assess the safety of surgery, and the lower the cardiovascular event rate, the higher the safety of surgery. The cardiovascular event rate in the beach chair position group (8.97% vs. 1.43%) was higher than that in the lateral decubitus position group, and the difference was statistically significant (*P* < 0.05), suggesting that compared with the lateral decubitus position, the beach chair position used for patients with rotator cuff injuries combined with shoulder adhesions undergoing shoulder arthroscopy may increase the risk of cardiovascular events, as shown in Table 4.

**Table 4:** Comparison of cardiovascular event rates between the two groups of patients.

Index	Lateral decubitus position group (*n* = 70)	Beach chair position group (*n* = 78)	*χ*² value	*P* value
Arrhythmia requiring intervention (*n*)	0	2		
Myocardial infarction (*n*)	0	2		
Angina pectoris (*n*)	0	1		
Hypotension (*n*)	0	1		
Postural hypotension (*n*)	1	1		
Total incidence (%)	1 (1.43)	7 (8.97)	4.108	0.043

## 4 Discussion

The rotator cuff consists of the tendons of the subscapularis, supraspinatus, infraspinatus, and teres minor muscles, and is shaped like a “cuff” that wraps around the anterior, superior, and posterior aspects of the shoulder joint. The tendons of these four muscles surround the shoulder joint, forming a “sleeve” like structure, which is figuratively called rotator cuff. Rotator cuff injury occurs when the soft tissue of these tendons is damaged (17). Shoulder joint adhesion is more common in patients with rotator cuff injury, due to the specificity of the injury site, often take surgical treatment, but the overall efficacy of surgery during the actual treatment can be reduced by the influence of extrinsic factors, including the surgical position is an important extrinsic factor (18). In this study, we found that the beach chair position of patients undergoing shoulder arthroscopy for rotator cuff injury combined with shoulder adhesion has a comparable effect on surgical efficiency and surgical trauma, and the improvement of shoulder function and mobility is similar to that of the lateral decubitus position, but it may cause fluctuations in blood pressure and heart rate, which may lead to an increased risk of cardiovascular events.

Regarding the selection and placement of the surgical position for patients undergoing shoulder arthroscopy for rotator cuff injuries combined with shoulder adhesions, different researchers have different opinions, with surgeons who prefer the beach chair position believing that the body is in a normal anatomical position, intraoperative anatomical structures are easy to localize, and the damaged structures and pinning position are easy to determine (19). However, proponents of the lateral decubitus position argue that the intraoperative scapular glenoid parallel to the floor when the patient is in the lateral decubitus position creates a new standard reference point (20). The present study showed that the differences in operative time, intraoperative bleeding, urine output, and fluid administration between the two groups were not statistically significant (*P* > 0.05), suggesting that the two positions have comparable effects on surgical efficiency and surgical trauma in patients undergoing shoulder arthroscopy for rotator cuff injuries combined with adhesions in the shoulder joint. When the surgery is performed in two positions, the surgeon can obtain a good surgical field of vision, which, together with modern hemostatic techniques and equipment, as well as standardized intraoperative fluid management as described in our anesthetic protocol, ultimately leads to a small difference in the surgical time, intraoperative bleeding, urine output, and fluid administration. There was also no statistically significant difference in the comparison of UCLA score, ASES score, forward flexion mobility, external rotation mobility and abduction mobility between the two groups of patients before and after surgery (*P* > 0.05), suggesting that the two groups of positions also had similar effects on the improvement of shoulder joint function and shoulder joint mobility. Continuous traction of the affected limb in the lateral decubitus position can better expose the subacromial space and the acromial side of the rotator cuff, which is conducive to the cleaning of the hyperplastic synovium in the subacromial space and the loosening of adhesion to the surrounding tissues, and at the same time, due to the clear exposure of the surgical field, it can reduce the damage to the normal tissues and reduce the postoperative re-adhesion, which is conducive to the early development of the patients' functional exercises after the operation and the promotion of the recovery of their shoulder joint function (21, 22). The beach chair position puts the shoulder joint in a relatively stable and easy-to-operate position, fully exposing the soft tissues around the shoulder joint, making it easy for the surgeon to manipulate instruments to loosen adhesions or deal with complex anatomical structures, thoroughly treating the lesion, and then improving the postoperative shoulder joint function (23, 24). Therefore, the postoperative shoulder joint function and mobility improvement of patients undergoing shoulder arthroscopy for rotator cuff injury combined with shoulder adhesions in both positions are basically the same.

The current study showed that the difference in systolic blood pressure, diastolic blood pressure, and heart rate before and after placement in the surgical position was higher in the beach chair position group than in the patients in the lateral decubitus position group, and the incidence of cardiovascular events was also higher than that in the patients in the lateral decubitus position group, and the difference was statistically significant (*P* < 0.05), suggesting that patients with shoulder arthroscopy with rotator cuff injury combined with shoulder adhesion may adopt the beach chair position to increase the incidence of cardiovascular events by causing hemodynamic fluctuations compared with the lateral decubitus position. Chillemi C et al. (25) found that shoulder arthroscopy in the beach chair position was prone to blood pressure and heart rate fluctuations, leading to adverse cardiovascular events, which is similar to the findings of this study. However, O'Neill C et al. (26) found that the beach chair position did not increase the incidence of postoperative cardiovascular events, and is a safer surgical position. The discrepancy between our findings and those of O'Neill C et al. may be explained by several important methodological and population differences. First, the study by O'Neill C et al. focused primarily on cerebrovascular accidents rather than the broader spectrum of cardiovascular events assessed in our study, including arrhythmias, myocardial infarction, angina, and hypotension. Second, their patient population was predominantly younger with a mean age of 52 years, compared to our elderly cohort with a mean age of approximately 65 years, which represents a population with inherently higher cardiovascular vulnerability and decreased compensatory mechanisms for hemodynamic changes. Third, differences in anesthetic management protocols, including the use of deliberate hypotensive anesthesia techniques and variations in fluid management strategies, may account for the different outcomes observed. Fourth, the definition and threshold for cardiovascular events may have differed between studies, with our study employing more stringent and comprehensive definitions that captured a wider range of hemodynamic complications (27).

The physiological mechanism underlying the hemodynamic fluctuations observed in the beach chair position can be explained by the effects of gravity on cardiovascular physiology. In the lateral decubitus position, the body is in a horizontal or slightly tilted state, the venous return is relatively stable, and the hemodynamic fluctuation is small, which can reduce the cardiovascular events caused by this (28, 29). In contrast, in the beach chair position, the patient's upper body is elevated (usually 60°–90°), and the lower limbs are dependent, which results in significant gravitational redistribution of blood volume. Specifically, the upright positioning causes blood to pool in the lower extremities and the splanchnic venous system due to the hydrostatic pressure gradient created by gravity. This venous pooling can result in a reduction of central blood volume by approximately 500–800 mL, significantly decreasing venous return to the right atrium. According to the Frank-Starling mechanism, this reduction in venous return directly decreases right ventricular preload, leading to decreased right ventricular stroke volume and subsequently reduced pulmonary blood flow. The diminished pulmonary venous return then decreases left ventricular preload, resulting in reduced left ventricular stroke volume and cardiac output, which can decrease by 15%–25% in the beach chair position compared to supine positioning (30). The baroreceptor reflex attempts to compensate for these changes through increased sympathetic tone, resulting in tachycardia and peripheral vasoconstriction. However, in elderly patients or those with impaired autonomic function, cardiovascular disease, or concurrent general anesthesia, these compensatory mechanisms may be blunted or delayed, leading to sustained hypotension and increased risk of end-organ hypoperfusion. Furthermore, the reduction in cerebral perfusion pressure due to the elevated head position can be particularly concerning, as the brain is positioned approximately 20–30 cm above the heart level, requiring additional perfusion pressure of approximately 15–22 mmHg to maintain adequate cerebral blood flow (31). These hemodynamic changes ultimately manifest as the observed differences in blood pressure and heart rate, and may in turn increase the incidence of various cardiovascular events (32, 33).

Based on our findings, we propose the following clinical decision-making guidance for position selection in patients undergoing shoulder arthroscopy for rotator cuff injury combined with shoulder adhesion. The lateral decubitus position should be preferentially considered for patients meeting any of the following criteria: age greater than 70 years, pre-existing cardiovascular disease (including coronary artery disease, heart failure with ejection fraction <50%, or history of myocardial infarction), uncontrolled hypertension (systolic blood pressure >160 mmHg or diastolic blood pressure >100 mmHg despite medication), diabetes mellitus with evidence of autonomic neuropathy, history of stroke or transient ischemic attack, ASA classification III or higher, or baseline orthostatic hypotension. For patients without these high-risk features, both positions may be safely utilized based on surgeon preference and anatomical considerations. When the beach chair position is selected, additional precautions should be implemented, including: gradual position changes with continuous hemodynamic monitoring, maintenance of mean arterial pressure within 20% of baseline values, consideration of lower limb compression devices to reduce venous pooling, adequate preoperative hydration, and immediate availability of vasopressor agents. Therefore, patients in the beach chair position have more obvious hemodynamic fluctuations and higher risk of cardiovascular events, which require close monitoring of blood pressure, heart rate during surgery to detect and treat any abnormalities in a timely manner; intraoperative adjustment of anesthesia medication and infusion rate to maintain hemodynamic stability as implemented in our standardized anesthetic protocol; elderly patients or patients with a history of cardiovascular disease should be careful to select the beach chair position and detailed risk assessment should be performed before surgery; postoperative monitoring of the patient's cardiovascular status should be continued so that any potential cardiovascular events can be detected and dealt with in a timely manner to ensure the safety of the patient's surgery.

However, there are still several limitations in this study that should be acknowledged. First, the sample size was relatively small (*n* = 148) and all patients were recruited from a single institution, which may limit the generalizability of our findings to other populations and healthcare settings. Although our *post-hoc* power analysis indicated adequate statistical power to detect significant differences in cardiovascular events, future multicenter studies with larger sample sizes would provide more robust evidence. Second, as a retrospective study, our analysis relied on existing clinical databases and medical records, which may have incomplete or missing data, potentially affecting the accuracy and reliability of the results. We cannot exclude the possibility of unmeasured confounders, such as variations in surgical technique or individual surgeon experience, that may have influenced the outcomes. To address this limitation, we have now included data on operator experience and duration of position maintenance in our baseline characteristics analysis, demonstrating comparable values between groups. Third, we did not assess cerebral perfusion status, cerebral blood flow, or intracranial pressure in our patients. This represents an important gap in our understanding of the neurological safety profile of different surgical positions, particularly given that cerebral hypoperfusion is a recognized concern with the beach chair position. Future prospective studies should incorporate neuromonitoring techniques, such as near-infrared spectroscopy or transcranial Doppler ultrasound, to evaluate cerebral oxygenation and blood flow patterns, as well as to assess the risk of cerebrovascular events in patients undergoing shoulder arthroscopy in different positions. Fourth, while we implemented standardized anesthetic protocols as described in our methods, subtle variations in anesthetic management between cases may have occurred and could potentially influence hemodynamic outcomes. Finally, our follow-up period was limited to 3 months postoperatively, which may not capture longer-term functional outcomes or delayed cardiovascular complications.

Despite these limitations, our study provides valuable insights into the hemodynamic and cardiovascular implications of surgical positioning in shoulder arthroscopy. The findings emphasize the importance of careful patient selection and position-specific risk assessment, particularly in elderly patients or those with pre-existing cardiovascular disease. To address the limitations identified in this study, future research should focus on conducting large-scale, multicenter, prospective randomized controlled trials that incorporate comprehensive neuromonitoring and longer follow-up periods. Additionally, studies should explore strategies to mitigate the hemodynamic effects of the beach chair position, such as gradual position changes, lower limb compression devices, or modified angles of elevation. Establishing strict data collection processes and quality control standards will be essential to improve the accuracy and comprehensiveness of the results, ultimately providing more reliable and substantial support for clinical decision-making regarding optimal surgical positioning in patients undergoing shoulder arthroscopy for rotator cuff injury combined with shoulder adhesion.

## 5 Conclusion

In this retrospective cohort study, the beach chair position in patients undergoing shoulder arthroscopy for rotator cuff injuries combined with shoulder adhesions had comparable effects on surgical efficiency and surgical trauma to the lateral decubitus position, and similar effects on shoulder function and mobility improvement to the lateral decubitus position, but it may lead to hemodynamic fluctuations and an increase in the incidence of cardiovascular events. These findings suggest that careful consideration of patient-specific factors, particularly cardiovascular risk profile, should guide the selection of surgical position in this population. Specifically, the lateral decubitus position should be preferentially considered for patients aged over 70 years, those with pre-existing cardiovascular disease, uncontrolled hypertension, or ASA classification III or higher, while both positions may be safely utilized in lower-risk patients based on surgeon preference and anatomical considerations.

## Data Availability

The raw data supporting the conclusions of this article will be made available by the authors, without undue reservation.
